# 20 years of the socioeconomic impact of atopic dermatitis and alopecia areata from around the globe

**DOI:** 10.1002/clt2.70061

**Published:** 2025-05-21

**Authors:** Katarina Stevanovic, Manuel Pereira, Ophélie Nguyen, Ingrid van Hofman, Cathrin Meesch, Torsten Zuberbier

**Affiliations:** ^1^ Institute of Allergology Charité – Universitätsmedizin Berlin, Corporate Member of Freie Universität Berlin and Humboldt‐Universität zu Berlin Berlin Germany; ^2^ Fraunhofer Institute for Translational Medicine and Pharmacology ITMP, Immunology and Allergology Berlin Germany; ^3^ GA^2^LEN Global Allergy and Asthma Excellence Network Berlin Germany

**Keywords:** alopecia areata, atopic dermatitis, disease costs, socioeconomic impact

## Abstract

Atopic dermatitis (AD) and alopecia areata (AA) represent chronic inflammatory diseases characterized by heterogeneous immune‐mediated mechanisms, including subtypes that may interconnect the two diseases, as well as other comorbidities. AD is globally recognized as the most common inflammatory skin disease and AA is an autoimmune disease, causing non‐scarring hair loss. In both diseases the quality of life (QoL) is decreased, out‐of‐pocket expenses on alternative therapies and camouflage endeavours is high, increased productivity loss/absenteeism at work or school, and high healthcare costs are significant. These diseases are not life threatening but result in a substantial socioeconomic impact, which so far has been difficult to quantify on the global scale. This qualitative review that includes literature published between 2004 and 2024 evaluates the current alignment between available healthcare resources and the comprehensive needs of these patients. Currently available data indicates that the socioeconomic impact of AD and AA is evidently high, meanwhile there is data lacking from most countries in Africa, Scandinavia and East Europe, the Middle East, South Asia, and parts of Latin America. Global studies with standardized methodology are necessary to assess the socio‐economic impact of these conditions.

## 1 INTRODUCTION

Atopic dermatitis (AD) and alopecia areata (AA) represent chronic inflammatory diseases characterized by heterogeneous immune‐mediated mechanisms, including subtypes that may interconnect the two diseases, as well as other atopic and non‐atopic comorbidities.^1^ AD is a highly pruritic, systemic inflammatory disease.^2^ It is globally recognized as the most common inflammatory skin disease and is more prevalent in childhood than adulthood.^3^ AA is an autoimmune disease, causing non‐scarring hair loss of the scalp, face, or body, prevalent in all sexes and can occur at any age.^4^ The clinical course of these diseases often follows unpredictable trajectories, with periods of relapse and remission, though severe disease is usually chronic. The co‐occurrence of AD with AA, and vice versa, is observed at a heightened prevalence compared to the general population.^5^ A multi‐centre, retrospective study showed that AD patients are at a 26‐fold risk of developing AA compared with healthy controls, and that up to 16% of adults with AD have AA.^6^ These conditions impose not only physical discomfort, marked by intolerable itching, impaired physical appearance, skin damage, and susceptibility to infection, but also lead to sleep disruption, diminish cognitive focus and energy levels, and undermine self‐esteem. Consequently, these diseases severely impact various aspects of daily life, such as social interactions, academic pursuits, and work. While AD and AA are not life threatening, they profoundly diminish the quality of life of affected individuals and their caregivers, leading to relentless pursuit of medical and over‐the‐counter remedies, very frequent visits to healthcare providers, and often precipitating social and professional absenteeism. Consequently, these conditions exert a substantial socioeconomic impact on patients and their immediate social and professional networks, as well as the healthcare system.

The Global Allergy and Asthma Excellence Network (GA^2^LEN) is the largest multidisciplinary network in the field of allergy and asthma research and clinical care, which was established in 2004. Within GA^2^LEN, the ADCARE group is dedicated to advancing expertise in AD. This sub‐network comprises clinical specialists and patient organisations, working together to conduct research and educational initiatives.

Herein, we present findings from a qualitative literature review spanning from 2004 to January 2024 that considered large cohort studies on AD and AA, reported in full articles and only in English, and searched in the PubMed literature database. Included reports were related to direct and indirect socioeconomic consequences and the quality of life for both patients and caregivers, using the main socioeconomic factors as outlined in previous similar analyses.^7^ Key points from reports that were included in this review are summarized in Table 1.

**TABLE 1:** Key points from included studies in this review on the socioeconomic impact of AD and AA, respectively, categorized by different aspects of impact.

Impact	Reported data on AD	Reported data on AA
Impaired sleep/Anxiety/Depression/QoL	• 3.6x higher chance of depression in AD patients versus healthy controls (Italy)^8^ • 43% adult AD patients experienced depression (Ireland)^9^ • 70.7% of AD patients experienced depression (USA)^10^ • Mean DLQI was 10.8 (moderate effect) in AD patients (28 countries worldwide)^11^	• 30% AA patients pursued a form of psychotherapy (USA)^12^ • 66.7% AA patients experienced depression (Nepal)^13^ • 41.8% of AA patients experienced depression (Japan)^14^ • Mean DLQI was 10.7 (moderate effect) in severe AA patients (Iran)^15^ • Significantly higher depression rates and new onset of depression prevalence in AA patients (UK)^12^
Out‐of‐pocket expenses	• 25% of parents spend up to €2300 annually for their child's AD (Ireland)^9^ • Mean annual cost is €461.1 for severe AD and €247.4 for moderate AD (France)^16^ • Mean annual cost is €927.12 (Western Europe)^17^	• Mean additional costs per patient were €1248 annually (Germany)^18^ • Mean additional cost per patient for medication was 390 USD (€357) annually (Japan)^14^ • Median annual expense on AA‐related products and services £840 (€994) per patient, £700 (€828) of which was on wigs (UK)^19^
Work absenteeism/presenteeism	• 24% adult and child patients missed 1–2 days of work or school, respectively, within 1 month (Ireland)^9^ • 13% patients missed 11 days or more per year due AD, and absenteeism was up to 3.85x higher with increasing AD severity (Western Europe)^17^	• 23.9% AA patients reported impaired presenteeism and 670 million USD was the nationwide cost due productivity loss (Japan)^14^ • Time off work certificates were more frequently issued to people with AA (13.0% within a year of diagnosis) than matched controls (7.9%) and AA patients were more likely to have a record of unemployment in the year after diagnosis (1.3% of cases of AA, 0.6% of matched controls)^20^
Healthcare costs	• Unadjusted annual health care costs in 2018 were $4979 (€4558) higher for adults AD patients versus healthy controls (USA)^21^ • Mean total direct and indirect costs per AD patient were €4331 annually (Hungary)^22^ • Mean annual direct costs per adult AD patient were €2963 higher versus healthy controls (Sweden)^23^	• Direct medical costs were 8557 USD (€7834) annually for AA patients, 1.3x higher than healthy controls, most of the increased costs were pharmacy costs related (USA)^24^

*Note*: All reported currencies were converted to the Euro (€) as well for ease of comparability using the Google convertor on March 11th, 2025.

Abbreviations: AA, alopecia areata; AD, atopic dermatitis; DLQI, Dermatology Life Quality Index; QoL, quality of life; UK, United Kingdom; USA, United States of America.

## 2 QUALITY OF LIFE IMPAIRMENT AND PSYCHOLOGICAL IMPACT

Living with inflammatory skin diseases, such as AD and AA, presents significant challenges for affected individuals and their caregivers. These conditions impact various aspects of daily life, leading to substantial psychological and behavioural changes, including lowered self‐esteem, social isolation, anxiety of flare ups, societal perception, depression, hopelessness, and even suicidal behaviour. The impact of these diseases on the quality of life is profound, affecting individuals of all ages. For young children, this often means bullying, exclusion, or isolation from social activities, while for adults, it can strain social and romantic relationships.^25^, ^26^, ^27^, ^28^, ^29^, ^30^ The summation of the entire impact, from disturbed daily activities such as stinging wounds while cutting fruit, or to a more extreme impact such as psychological disturbances, gives a strongly impaired overall quality of life for these patients, highlighting the critical need for effective management strategies.

Over 30% of AD or AA sufferers report depression and anxiety across the globe, which is significantly higher than in healthy controls.^10^, ^13^, ^31^ These studies report that sleep quality is particularly compromised in AD patients, exacerbating stress, reducing productivity, impairing the immune system, and perpetuating the cycle of these inflammatory diseases.

### 2.1 AD

A comprehensive UK‐based study involving child AD patients (*N* = 4938) and healthy controls (*N* = 9050) found that although total sleep duration was similar, children with active AD reported significantly worse sleep quality and had nearly 50% higher odds of experiencing sleep disturbances, especially those with comorbid asthma or allergic rhinitis.^32^ In Ireland, parents of children with AD (*N* = 240) reported that their child had interrupted sleep on a weekly basis (89%), and avoided social activities (39%) and exercise (34%) because of their AD.^9^ The same study also analysed adult AD patients (*N* = 215) in which 5% described interrupted sleep, 70% reported social anxiety, 65% avoided exercise and sports, 52% avoided social activities, 52% avoided sexual intimacy and 43% were depressive.^9^ A study conducted in the United States reported that adults with AD had lower Short Form‐6 Dimensional (SF‐6D) scores compared with healthy individuals (0.69 vs. 0.79).^33^ The reduction in SF‐6D scores was particularly pronounced in individuals with AD who also had atopic comorbidities.^33^

### 2.2 AA

Considering AA in this regard, the results are similar to those of AD. Analysis of 400 AA patients in Japan revealed lower Short Form Health Survey 36 Item Version 2.0 (SF‐36v2) subscale scores for patients with AA specifically for mental health (45.7 ± 10.1 points), social functioning (45.8 ± 10.9 points), vitality (46.2 ± 9.8 points), and role emotional (46.9 ± 11.6 points) as compared to the Japanese population norms of 50 ± 10 points each.^34^ Multivariate linear regression of the results of the DLQI questionnaire showed that hair loss range, age, comorbidities, and depression significantly worsened DLQI scores.^34^ Similarly, a study in Iran with 176 AA patients revealed that the mean DLQI scores in the severe and mild groups were 10.7 ± 7.5 and 5.4 ± 6.8, respectively.^15^ The score difference was significantly higher in the severe group reflecting a more impaired quality of life, and particularly this group consisted mainly of women.^15^

The results of these surveys demonstrate that a significant decrease in the quality of life, increased sleep impairment, and high incidences of anxiety and depression are observed globally in patients with AD and AA in comparison with mild cases or healthy controls. Hence, emphasis on mental health is crucial for the management of these diseases.

## 3 OUT‐OF‐POCKET EXPENSES

Given the significant daily life hurdles and psychological difficulties associated with AD and AA, it is only to be expected that the patients would seek any possible means to find relief or conceal their issues. This includes over the counter medications, cosmetic camouflage items, or alternative therapies not covered by health insurance.

### 3.1 AD

In the previously mentioned study from Ireland involving parents of AD patients and adult AD patients, 52% adult patients reported that they cannot always afford treatments for their AD and must cut back on their treatment expenses.^9^ For treatment, 28% of adults spend under €25, 35% spend €25–50, 25% spend €50–100, 10% spend €100%–150%, and 2% spend over €150 on over the counter (OTC) treatments per month. In the same study, 58% of parents indicated that they needed to cut back on household spending to be able to afford their child's treatments. For their child's AD treatments, 34% of parents spend under €25, 30% spend €25–50, 24% spend €50–100, 7% spend €100%–150% and 3% spend over €150 on OTC. Regarding prescription treatments (e.g. topical steroids, oral steroids, other systemic treatments), 55% of adult respondents spend over €25, 20% spend €25–50, 15% spend €50–100, 6% spend €100%–125% and 4% spend over €125. Figures are similar among the parent group for costs of their child's monthly prescription costs, such as 53% of parents spend under €25, 19% spend €25–50, 17% spend €50–100, 6% spend €100%–125% and 5% spend over €125. Besides OTC and prescription treatments, patients with AD often seek alternative medical therapy, with up to 40% of adult patients and 35% of parents using alternative treatments to manage their or their child's AD. The majority, 55% of the adult patients and 65% of parents, spend up to €50/year, and 10% report spending over €200/year on alternative therapies for their eczema. These data suggest that one‐quarter of those surveyed spend up to €2300 annually on OTC treatments, prescription treatments, alternative treatments and doctor's fees because of their or their child's AD.

In a study conducted in France including 1024 AD patients, the mean annual out‐of‐pocket cost per person was €462.1 for severe AD and €247.4 for moderate AD.^16^ Emollients were the most used product, with 74.4% of patients using them at an average out‐of‐pocket cost of €151.4. Out‐of‐pocket costs increased significantly with the severity of the condition, with 27% of patients with severe AD purchasing specially textured clothes compared with 19% of patients with moderate AD.

A study involving 1189 AD patients across Western Europe (Czech Republic, Denmark, France, Germany, Italy, the Netherlands, Spain, Sweden and the UK) confirmed that extra spending on everyday necessities is common among patients with AD.^17^ While only 5% of respondents did not report extra expenses, most AD patients spend on average an additional €927.12 per year on healthcare. The mean extra spending was €77.26/month, with emollients and moisturizers accounting for the highest monthly costs (€27.63), followed by medication (€17.74) and doctor and hospital visits (€8.68). Occasional additional mean monthly costs for some patients also included phototherapy (€8.48), bandages (€7.12), and travel expenses (€5.69).

### 3.2 AA

In the case of AA, a worldwide study with 675 AA patients reported that 60.1% of participants had out‐of‐pocket spending in at least 4 categories of expenses, with the greatest percentage spending on hair salon appointments (81.8%) and vitamins and/or supplements (67.7%).^35^ The median yearly spending, 450 USD (€411), was highest for headwear or cosmetic items such as hats, wigs, and makeup. Most participants rated their financial burden of AA as moderately (31.7%) or seriously (25.2%) burdensome. To manage these expenses, participants reported using their savings (41.3%), as well as cutting down on expenses (40.3%), recreational activities (36.7%), or spending on food or clothes (33.9%). Participants reported a median spending of 1354 USD (€1239) annually.

Globally, out‐of‐pocket costs vary, likely due to differences in national economic standards, insurance policies in different countries, and individuals' willingness to spend on self‐paid help. However, it can be concluded that out‐of‐pocket costs contribute significantly to the management difficulties of these diseases. The financial burden reduces patients' quality of life and further indirectly propagates the diseases by contributing to stress.

## 4 WORK ABSENTEEISM AND PRESENTEEISM

### 4.1 AD

The impact of AD on patients extends beyond quality of life and psychological stress to include significant financial impact. In turn, this also affects their education and career prospects, leading to additional psychological and economic stress on the patients, and eventually on the tax‐paying society through work absenteeism (i.e. frequent absence from work/school) and presenteeism (i.e. being at work/school but not fully productive). These costs also fall in the category of indirect disease costs for the economy.

A study in Europe and the United States examined the effect of AD severity on work productivity and daily activities among adults.^36^ The study included 1098 respondents with moderate‐to‐severe AD and 134 with mild AD. Findings indicated that as AD severity increased, so did the negative impact on work productivity. Patients with mild, moderate, and severe AD reported losing an average of 2.4 h/week, 9.6 h/week, and 19.0 h/week, respectively. In a similar study including patients from across Western Europe, 57% of AD patients missed at least one day of work in the previous year, with 26% missing at least 1 week, and 13% missing 11 days or more.^17^

A study in Taiwan explored the impact of AD on work productivity and daily activities, classifying results by disease severity.^37^ One‐third of employed participants reported missing work due to AD, and 88.5% of the remaining two‐thirds reported reduced work effectiveness. Overall work impairment was 1.8 and 2.6 times greater in those with moderate and severe AD, respectively, compared with those with mild AD. Daily activity impairment was also increased, at 1.5 and 2.0 times higher for moderate and severe AD, respectively. These impairments correlated strongly with AD severity scores (SCORAD).

### 4.2 AA

For AA patients in Japan, work productivity was significantly impacted by presenteeism (23.9% ± 25.7%), while absenteeism remained low (0.9% ± 2.8%).^14^ In the UK, a study with 829 AA participants showed that 21% had been signed off work due to AA since their diagnosis.^38^ Over the past week, 10.5% of employed participants missed more than 1 hour of work due to AA. Of those claiming work‐related benefits, 33 linked their claims to AA. Despite these challenges, the average impact of AA on work productivity over the past week was minimal, with those recently diagnosed reporting the greatest loss in productivity. A Western Europe‐wide study reported that 18.2% of AA patients experienced a median annual income loss of 500 USD (€457) due to missed work.^21^

In summary, both AD and AA significantly impact work absenteeism and presenteeism, strongly correlating with disease severity. Patients with more severe conditions experience greater productivity losses and daily activity impairments, leading to substantial personal and societal economic costs. Addressing these impacts requires targeted interventions to support affected individuals in managing their conditions and maintaining their professional and personal lives.

## 5 HEALTHCARE COSTS

Another substantial impact of these diseases is on the healthcare system.

### 5.1 AD

Adults with AD utilize more outpatient services, pharmacy services, and short‐term disability benefits than those without AD.^21^ In a 2018 study in the United States, it was shown that unadjusted annual healthcare costs were 4979 USD (€4558) higher for adults with AD (14603 USD (€13369)) compared to controls (9624 USD (€8811)), primarily due to outpatient services and pharmacy costs.^21^ A limitation of this study is the potential underrepresentation of patients with mild AD, who may not seek medical treatment.

A Swedish study examined 195,719 paediatric, 34,717 adolescent, and 107,774 adult AD patients and compared them with age‐matched controls.^23^ It found that annual mean per‐patient direct healthcare costs in the first year post‐diagnosis were €941 and €1,259, €816 and €1,260, and €1583 and €2963 higher in paediatric, adolescent, and adult patients with moderate‐to‐severe and severe AD, respectively. Indirect costs for caring for sick children were €69 and €78 higher per moderate‐to‐severe and severe AD patient, respectively. For adults, the indirect costs were €148 and €263 higher for moderate‐to‐severe and severe AD patients, respectively. Overall, the societal economic cost for AD was €351 million and €96 million higher for patients with moderate‐to‐severe and severe AD, respectively, compared with controls.

### 5.2 AA

In the USA, a study of 14,340 AA patients versus 42,998 controls revealed higher healthcare resource utilization and higher adjusted total all‐cause mean medical costs for AA patients (8557 USD (€7833) versus 6416 USD (€5873); *p* < 0.0001).^24^ This increase was due to higher inpatient costs, emergency department visits, ambulatory visits, prescriptions, and other costs (such as durable medical equipment and home healthcare). Mean ambulatory costs were 3640 USD (€3240) for AA patients compared to 2062 USD (€1887) for controls, and mean pharmacy costs were 3287 USD (€3008) versus 1843 USD (€1686) (*p* < 0.0001 for both). Immunologic agents accounted for 50% of the difference in pharmacy spending, while integumentary system surgeries accounted for 9.5% of the difference in ambulatory costs.

In summary, both AD and AA significantly increase healthcare utilization and costs, primarily driven by outpatient services, pharmacy needs, and the management of comorbid conditions. Addressing these diseases' impacts on the healthcare system requires comprehensive strategies to manage these conditions effectively and reduce associated economic impact.

## 6 CONCLUSION

**FIGURE 1:**
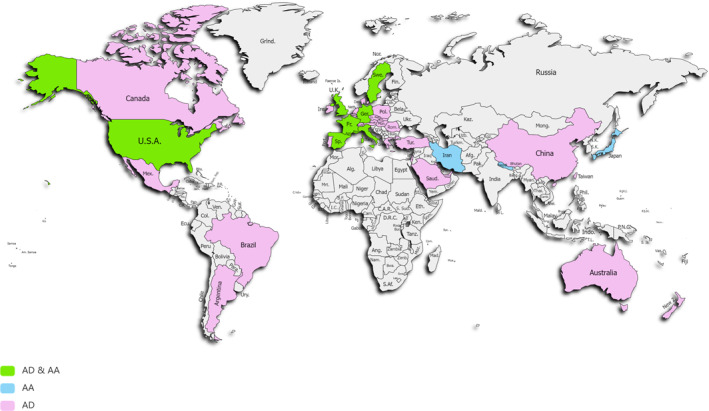
World map depicting data coverage included in this qualitative literature review, colour referring to data coverage on AD, AA, or both, respectively. AA, alopecia areata; AD, atopic dermatitis.

This qualitative review analysed global data from the past 20 years on the socioeconomic impact of chronic inflammatory diseases such as AD and AA. Figure 1 shows the geographical distribution of available data in the included literature. Larger cohort studies were prioritized to enhance data robustness, although efforts were made to maximize global coverage. Despite substantial data on AD and/or AA across regions, large data gaps remain in Africa, Eastern Europe, the Middle East, South Asia, and parts of Latin America, highlighting areas in need of further research. Initiatives like AWARE 1 have acknowledged this gap by conducting an international survey quantifying the epidemiology of adult patients with AD within these underrepresented regions.^39^ The Global Burden of Disease (GBD) study revealed that AD ranks 15th among all nonfatal diseases and has the highest disease burden among skin diseases as measured by disability‐adjusted life‐years (DALYs), a measure for the difference between living a life in perfect health and living with a disease.^40^ In Europe, the countries with the highest AD‐related DALY rates are Sweden, the UK, and Iceland, whereas Uzbekistan, Armenia and Tajikistan ranked lowest.^40^ Also, low disability weights were reported in China, which could potentially be due to underdiagnosis.^41^ Such metrics and high‐quality data are crucial in guiding public health strategies to address the nonfatal burden of diseases such as AD and AA.

**FIGURE 2:**
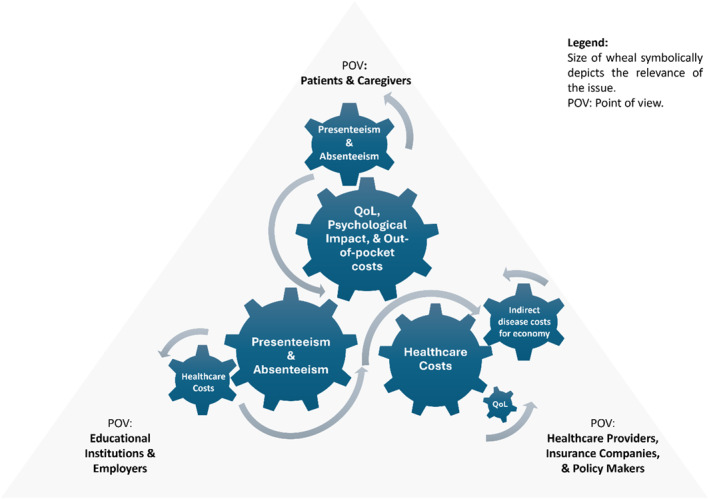
An illustrative interplay of different factors contributing to the socio‐economic impact of AD and AA shown from the perspective of three major stakeholders. AA, alopecia areata; AD, atopic dermatitis; QoL, quality of life; POV, point of view.

**FIGURE 3:**
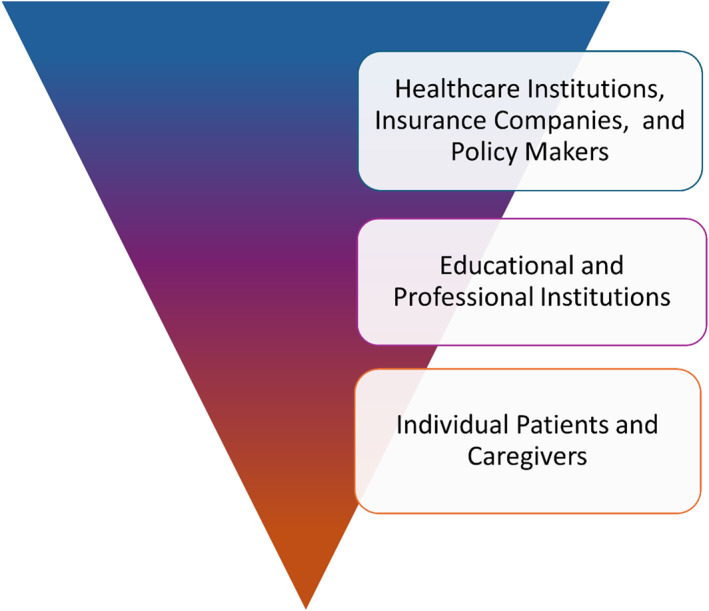
An illustrative inverted pyramid scheme indicating the number of people impacted in the different stakeholder layers by the socio‐economic impact associated with AD and AA. AA, alopecia areata; AD, atopic dermatitis.

The key factors influencing the socioeconomic impact of AD and AA include the diminished quality of life, psychological disorders, out‐of‐pocket treatment expenses, and work or school absenteeism and presenteeism, and healthcare costs. These individual factors perpetuate an interconnected clockwork mechanism impacting patients, caregivers, as well as local and broader economic systems (e.g., insurers, healthcare providers, and governments), as illustrated in Figure 2. Each stakeholder experiences and prioritizes these burdens differently based on their perspective and needs. The complexity of this issue is not unique to these diseases but is common when various stakeholders are involved. However, AD and AA stand out due to their high global prevalence, likely underestimated because of incomplete data. Despite not posing an immediate threat to life, these diseases are often neglected in healthcare reforms. Nevertheless, they have a substantial impact on quality of life and economic factors at multiple levels, affecting a vast range of individuals and stakeholders ‐ from those directly suffering from the conditions to policymakers, as illustrated in Figure 3.

To mitigate this impact, one approach would be for all stakeholders to implement specific measures. Patients should be educated and encouraged to use disease tracking tools. Physicians need regular updated training on treatments and implementation of integrated care pathways (e.g., AD ICPS^2^, ^42^), as well as transparency in healthcare costs with the patients and caregivers. Employers could provide flexible working arrangements, including remote work, and offer access to support services. Regulatory agencies, governments, and insurers should expand access to novel therapies.

The challenge of comparing cost data across studies stems from inconsistent methodologies in collecting and analysing such data. Beyond financial metrics, evaluating the sociological impact is also complex, as perceptions of impact about such diseases vary across countries and cultures. Standardized, comparable data are crucial for thorough patient education and informing pharmaceutical companies, healthcare authorities, and insurance policies. Such data would enable the development of consistent treatment protocols and integrated care pathways, ensuring that economic and sociocultural factors are considered when crafting comprehensive healthcare strategies.

## AUTHOR CONTRIBUTIONS

**Katarina Stevanovic**: conceptualization, investigation, writing – original draft, methodology, visualization, writing – review and editing, formal analysis, supervision, funding acquisition. **Manuel Pereira**: conceptualization, methodology, writing – review and editing, supervision. **Ophélie Nguyen**: formal analysis and data curation. **Ingrid van Hofman**: project administration, funding acquisition. **Cathrin Meesch**: funding acquisition, project administration. **Torsten Zuberbier**: conceptualization, supervision, writing–review and editing, funding acquisition, data curation, formal analysis.

## CONFLICT OF INTEREST STATEMENT

**Manuel Pereira** has received research funding from Almirall; is an investigator for Allakos, Celldex Therapeutics, Incyte, Sanofi and Trevi Therapeutics; and has received consulting fees, speaker honoraria and/or travel fees from AbbVie, Beiersdorf, Celltrion, Eli Lilly, GA2LEN, Galderma, Menlo Therapeutics, Novartis, P.G. Unna Academy, Sanofi, StreamedUP and Trevi Therapeutics. **Torsten Zuberbier** reports honoraria for lectures from Amgen, AstraZeneca, AbbVie, ALK ‐Abelló, Almirall, Astellas, Bayer Health Care, Bencard, Berlin Chemie, FAES Farma, HAL Allergie GmbH, Henkel, Kryolan, Leti, L'Oreal, Meda, Menarini, Merck Sharp & Dohme, Novartis, Nuocor, Pfizer, Sanofi, Stallergenes, Takeda, Teva, UCB, and Uriach; Fees for industry consulting were received from Abivax, Almirall, Blueprint, Celldex, Celltrion, Novartis, and Sanofi; in addition he declares non‐paid organizational affiliations: Committee member, “Allergic Rhinitis and its Impact on Asthma” (ARIA), Member of the Board, German Society for Allergy and Clinical Immunology (DGAKI), Head, European Centre for Allergy Research Foundation (ECARF), President, Global Allergy and Asthma Excellence Network (GA^2^LEN), and Member, Committee on Allergy Diagnosis and Molecular Allergology, World Allergy Organisation (WAO). **Katarina Stevanovic, Ophelie Nguyen, Ingrid van Hofman, and Cathrin Meesch** declare no conflicts of interest.

## Data Availability

Data sharing not applicable to this article as no datasets were generated or analysed during the current study.
